# Physical Activity Status and Related Factors of Lung Transplant Patients Within One Year After Surgery: A Single‐Center Observational Study

**DOI:** 10.1155/carj/5036015

**Published:** 2026-08-24

**Authors:** Xin Chen, Xing He, DingDing Zhang, JianQiong Gao, Tian Xu, XiangLing Yue, ChunHan Cui

**Affiliations:** ^1^ Department of Pulmonary and Critical Care, Sichuan Provincial People’s Hospital, School of Medicine, University of Electronic Science and Technology of China, Chengdu, China, uestc.edu.cn; ^2^ Department of Pulmonary and Critical Care Medicine, West China Hospital, Sichuan University, Chengdu, China, scu.edu.cn; ^3^ State Key Laboratory of Respiratory Health and Multimorbidity, West China Hospital, Sichuan University, Chengdu, China, scu.edu.cn; ^4^ Department of Medicine, School of Medicine, Sichuan Provincial People’s Hospital, University of Electronic Science and Technology of China, Chengdu, China, uestc.edu.cn; ^5^ Department of Hepatobiliary and Pancreatic Surgery, Sichuan Provincial People’s Hospital, School of Medicine, University of Electronic Science and Technology of China, Chengdu, China, uestc.edu.cn

**Keywords:** lung transplantation, postoperative activity ability, related factors

## Abstract

**Background:**

After undergoing lung transplantation, patients’ mobility is closely associated with postoperative survival outcomes. This study aimed to assess the physical activity levels of lung transplant recipients at 1‐year postsurgery and identify its influencing factors.

**Methods:**

A prospective follow‐up evaluation was used to investigate the relationship between preoperative, intraoperative, and postoperative clinical factors and functional recovery outcomes. The Duke activity status index (DASI) score was evaluated at 1 year after surgery. SPSS software was used for statistical analysis.

**Results:**

Thirty lung transplant recipients were enrolled, with 27 males and 3 females. The age ranged from 23 to 72 years. The mean DASI score at 1 year postoperatively was 23.84 ± 13.66 points. Univariate analysis showed that age greater than 60 years was a relevant factor influencing DASI at 1 year postoperatively in lung transplant recipients (*t* = 3.562, *p* = 0.002). Multivariate analysis indicated that factors such as being over 60 years old (*β* = −0.476, *p* = 0.010), having a lower preoperative body mass index (BMI) (*β* = 0.592, *p* = 0.003), and a shorter hospital stay in the ICU (*β* = 0.480, *p* = 0.004) might be associated with a decline in postoperative activity ability of lung transplant patients.

**Conclusion:**

This suggests that patients over 60 years of age should be prioritized for postoperative mobility management. Among the various aspects of management for lung transplant recipients, avoiding a low preoperative BMI may be more beneficial for improving their long‐term postoperative mobility. These findings apply primarily to male lung‐transplant patients, and the generalizability of the results needs to be validated in larger cohorts.

## 1. Introduction

Lung transplantation is internationally recognized as one of the effective treatments for end‐stage lung diseases, and more than 70,000 lung transplants have been performed worldwide [1]. Although the 1‐year survival rate after lung transplantation is increasing, the survival rate of recipients in the second year after surgery is declining, and the 5‐year survival rate of approximately 70% after surgery is still lower than that of other solid organ transplants [1]. This is mainly related to more than a dozen kinds of postoperative complications that have a high incidence and cause serious harm [2]. Infection after lung transplantation is the most common cause of death in the first year after transplantation and may occur in up to 75% of lung‐transplant recipients [3]. The incidence of acute kidney injury was found to be more than 60%, and airway complications were also observed in 12.4% of cases [4]. Postoperative complications significantly increase the risk of death among lung transplant recipients.

Studies have found that postoperative exercise resulted in an average of four fewer complications and improved health‐related quality of life in lung transplant recipients compared with those who did not exercise [5, 6]. Every 0.3 improvement in the Lung Transplant‐Valued Life Activities Scale reduced a patient’s risk of death by 19% [7]. These findings indicate that postoperative patient mobility was closely related to their prognosis. However, postoperative mobility in lung transplant recipients continues to decline during the first year [8]. Currently, the long‐term postoperative activity status of Chinese lung transplant recipients and its influencing factors are not fully elucidated [9]. Therefore, this study employed the Duke Activity Status Index (DASI), which has been found to be more effective in predicting the degree of physical activity in patients with advanced lung diseases in recent years, to assess the activity ability of lung transplant recipients 1 year postoperatively [10]. The study aimed to analyze the related factors and provide a reference for enhancing the quality of long‐term survival and improving the survival rate of lung transplant recipients.

## 2. Patients and Methods

### 2.1. Patients

Patients who underwent lung transplantation in a general hospital in Southwest China from August 1, 2018, to April 1, 2024 were consecutively enrolled. The inclusion criteria were as follows: (1) age > 18 years; (2) first lung transplantation. Exclusion criteria were as follows: (1) combined multiple organ transplantation; (2) patients with limb movement disorder before the operation; (3) continuous use of sedative drugs for more than 3 days; (4) patients with a disturbance of consciousness; (5) survival time of less than 1 year.

### 2.2. Methods

In this study, multimodal data potentially associated with postoperative mobility in lung transplant recipients before, during, and after surgery were collected. The study was approved by the ethics committee of Sichuan Provincial People’s Hospital (No.: 2025188). Following written informed consent of patients, anthropometric measurements (height/weight) were obtained through standardized clinical assessments. Clinical variables including surgical parameters, comorbidity profiles, and Barthel Index (BI) scores were extracted from institutional electronic health records. Postoperative functional recovery was tracked through: (1) nursing staff documentation of predefined mobility milestones (e.g., ambulation > 5 m from bed and self‐care activities) using time‐stamped case report forms; (2) caregiver‐maintained activity logs for patients after discharge; and (3) weekly researcher‐administered telephone surveys verifying event chronology. The DASI was administered at 12‐month follow‐up via in‐person or telemedicine platforms. The termination criteria of the study included (1) mortality events; (2) sustained sedation (> 72 consecutive hours); and (3) inaccurate recording of the occurrence time of the investigation event by the caregiver or the patient.

All patients received standardized rehabilitation training during their postoperative hospitalization. Respiratory training and upper and lower limb training were carried out by a multidisciplinary team of rehabilitation therapists, respiratory doctors, nutritionists, and nurses. All patients began rehabilitation on the second day after surgery, and the rehabilitation therapists customized personalized rehabilitation programs according to the patient’s muscle strength, condition, and willingness. All patients participated in rehabilitation throughout the hospitalization (ICU and general ward). The rehabilitation therapist conducts bedside rehabilitation exercises at least once a day according to the individualized rehabilitation plan. The duration of the exercises is 30–60 min. These exercises include, but are not limited to, passive limb training, upper and lower limb exercises using equipment, breathing training, ankle pump movements, double upper limb muscle strength training using grip balls or elastic bands, sitting exercises, and bedside standing. Under the guidance of the rehabilitation therapist, the responsible nurse is responsible for urging the patient to increase the exercise frequency based on the patient’s tolerance level to achieve the most ideal rehabilitation exercise intensity. After discharge, patients who are still unable to walk to the bedside will be transferred to rehabilitation institutions to continue their rehabilitation process; other patients will go home and not participate in outpatient rehabilitation treatment.

### 2.3. Assessment Instruments

#### 2.3.1. Clinical Profile Questionnaire

Developed through a systematic literature review of mobility determinants in lung transplant recipients, this instrument captured multidimensional predictors including anthropometric measures, surgical parameters, and comorbidity profiles.

#### 2.3.2. BI

The scale was developed by Mahoney and Barthel [11] and is often used to assess the self‐care ability of hospitalized patients. It includes bathing, eating, dressing, grooming, toileting, stool control, urination control, bed and chair transfer, walking on the ground, and going up and down stairs. The patients were divided into four levels according to whether they needed help and the degree of help needed: 15, 10, 5, and 0 points. The total score was 100 points. The corresponding scores of complete dependence, severe dependence, moderate dependence, and mild dependence were as follows: 0–20, 21–60, 61–90, and 91–99, respectively, with 100 being completely self‐care.

#### 2.3.3. DASI

The scale includes 12 items such as personal care ability, walking ability, housework ability, sexual behavior ability, recreational activities, and physical activity ability. If the activity intensity specified in the scale cannot be achieved, a score of 0 will be given. If it can be achieved, points will be assigned based on the specific energy expenditure associated with the physical activity (ranging from 1.75 to 8 points). The maximum possible score is 58.2 points. The score can be used to estimate the patient’s peak oxygen uptake (VO_2_peak), VO_2_peak = 0.43 × (DASI+9.6), and the patient’s metabolic equivalent of task level (METs) can be obtained (METs = VO_2_peak/3.5). The higher the score, the stronger the patient’s activity ability. There were relevant studies that have used DASI as an evaluation tool for the postoperative activity status of patients [12, 13]. In this study, DASI was used as an outcome index to evaluate the postoperative mobility status of patients.

#### 2.3.4. Sample Size Calculation

The empirical sample size estimation method was used, and the estimated sample size was 5 times the number of potential candidate parameters included; 4 candidate parameters were calculated. The minimum sample size was designed as 20 to meet the needs of statistical analysis [14].

#### 2.3.5. Statistical Analysis

SPSS 26.0 statistical software was used to analyze the data. The Shapiro–Wilk test was conducted to test the normality of all measurement data. Measurement data conforming to a normal distribution were expressed as the mean ± standard deviation and analyzed by the independent‐samples *t*‐test or one‐way analysis. The measurement data that did not meet a normal distribution were analyzed by the Wilcoxon signed‐rank test or the Kruskal–Wallis test, and were expressed as the median and interquartile range. Enumeration data were expressed as the number of cases and the constituent ratio (%), and the chi‐square test was used for statistical analysis. Pearson or Spearman correlation analysis was used to analyze the related factors of the Duke performance status index. Collinearity diagnosis was used to evaluate the correlation between independent variables. If the tolerance was less than 0.1 or the variance inflation factor (VIF) was ≥ 5, it was considered that there was a serious multicollinearity problem, and the multiple linear regression model was rejected. A *p* value of less than 0.05 was considered statistically significant.

In this study, 50% of lung transplant recipients were over 60 years old. In order to determine whether there were differences in the characteristics affecting postoperative mobility between these two groups, we analyzed the differences in preoperative BMI, postoperative hospital stay, and BI score between lung transplant recipients aged 60 years and over and those under 60 years old. The results showed that their baseline characteristics were consistent and did not bias the results (Supporting Table 1).

## 3. Results

### 3.1. Patient Characteristics

A total of 30 lung transplant patients were enrolled in the study, of whom 3 patients worked at home, 1 had returned to school, 22 had retired, and 4 patients over the age of 50 had chosen not to work. More than 95% of the patients were male, and more than half of the patients were diagnosed with chronic obstructive pulmonary disease (COPD). None of the posttransplant recipients experienced rejection in the first year after transplantation. Four patients did not receive extracorporeal membrane oxygenation (ECMO) after surgery. The BMI was below normal in 33.3% of the patients, overweight in 16.7%, and normal in 50%, with a median and range of 19.4 (10.4,27.4) kg/m^2^. The proportion of patients over 60 years old was 50%. The mean preoperative BI score of the recipients was 61 (Table 1).

**TABLE 1:** Patient characteristics.

Event	Example (*n* = 30)	Mean ± standard deviation/median (quartiles)/percentage (%)
*Preoperative*		
Age	30	60 [53,66]
Sex		
Male	27	90
Female	3	10
Primary disease	15	50
COPD^a^	14	46.7
Interstitial lung fibrosis	1	3.3
Refractory pneumothorax^∗^		
Ventilatory support	7	23.3
Noninvasive ventilator	1	3.3
Invasive ventilator	22	73.3
Nasal conduit		
BI scores	30	61.2 ± 21.9
Surgical procedures	27	90
Dual lung transplantation	3	10
Single lung transplantation		

*Postoperative*		
Postoperative complications in patients	30	100
ICHP^b^	6	20
Pleural effusion	6	20
Hypoproteinemia	2	6
Chronic renal insufficiency	2	6
Venous thrombus	2	6
Pneumothorax	2	6
Skin soft‐tissue infection	2	6
Pulmonary heart disease	1	3
Acute liver injury		
Drug‐resistant bacterial infection		
Yes	19	63.3
No	11	36.7
Duration of ECMO^c^ support (*h*)	26^g^	26.5 [20.5, 40.5]
Length of mechanical ventilation (*h*)	30	49 [30.8, 621]
ICU^d^ length of hospitalization (*h*)	30	129 [72, 204]
Length of stay in general ward (*h*)	30	1016.5 [660, 1512]
Total length of hospitalization (*h*)	30	1200 [781.5, 1728]

*Length of time between the end of the operation and the time of the event*		
Initiation of rehabilitation (*h*)	30	129 [79.3, 270]
Removal of gastric tube (*h*)	30	171.5 [85.3, 457.3]
BI scores > 60 (*h*)	30	741 [501.5, 1434]
> 5 m away from the bed (*h*)	30	360 [228, 1014]
> 30 min out of bed (*h*)	30	492 [276, 1242]
Self‐washing (*h*)	30	540 [288, 1068]
Self‐feeding (*h*)	30	504 [234, 1008]
Self‐bathing (*h*)	30	1236 [810, 1776]
Walking aiding (*h*)	30	612 [288, 1038]
Independent walking (*h*)	30	780 [360, 1554]
DASI	30	23.84 ± 13.66
VO2^e^	30	19.37 ± 6.20
METS^f^	30	5.53 ± 1.77

^a^Chronic obstructive pulmonary disease.

^b^Immunocompromised host pneumonia.

^c^Extracorporeal membrane oxygenation.

^d^Intensive care unit.

^e^VO_2_peak = 0.43 × DASI + 9.6.

^f^Metabolic equivalent of task (METS) = VO_2_peak/3.5.

^g^Four people did not use ECMO.

^∗^A patient with refractory pneumothorax caused by Castleman’s disease.

### 3.2. Activity Status of Lung Transplant Recipients

The results of univariate analysis showed that lung transplant recipients aged ≥ 60 years had significantly lower Duke Index scores than those aged < 60 years (*p* = 0.002), whereas preoperative mode of ventilatory support, BMI, surgical modality and duration, primary morbidities, preoperative BI Scores, and multidrug‐resistant bacterial infections in the postoperative period were not statistically significant in the Duke Activity Index scores of recipients at 1 year postoperatively (Table 2).

**TABLE 2:** Differential analysis of activity status in lung transplant recipients.

Event	Number of examples	Duke index score	Statistical value	*p*
Age				
< 60 years	15	31.04 ± 13.11	t = 3.357	0.002
≥ 60 years	15	16.64 ± 10.19		
Preoperative mechanical ventilation				
Yes	8	22.95 [8.11,41.95]	Z = −0.189	0.850
No	22	18.95 [15.33,34.70]		
Preoperative BMI^a^ kg/m^2^				
< 18.5	10	17.44 ± 11.74	H = 3.205	0.202
18.5–23.9	15	24.90 ± 11.23		
24–27.9	5	33.45 ± 19.5		
Surgical Procedures				
Single lung transplantation	3	24.20	Z = −0.068	0.946
Dual lung transplantation	27	23.80 ± 14.21		
Primary disease				
COPD^b^	15	20.84 ± 13.10	t = −1.287	0.209
Interstitial lung fibrosis	14	27.40 ± 14.35		
Refractory pneumothorax	1	NA		
Surgical time				
≥ 6 h	22	23.44 ± 14.75	t = 0.264	0.794
< 6 h	8	24.95 ± 10.89		
The number of hospitalizations within 1 year after the surgery				
≤ 1	18	13.08 [10.14, 37.95]	H = 1.010	0.603
2–4	8	18.95 [14.20, 36.08]		
> 4	4	18.95 [17.20, 34.70]		
Postoperative drug‐resistant bacterial infections				
Yes	19	22.14 ± 12.48	t = 0.891	0.380
No	11	26.77 ± 15.69		
Preoperative BI scores				
≤ 60	11	18.32 ± 12.30	t = −1.741	0.093
> 60	19	27.03 ± 13.69		

^a^Body mass index based on the Chinese standards.

^b^Chronic obstructive pulmonary disease.

^c^Excluding hospitalization for lung transplant surgery.

### 3.3. Factors Associated With Activity Status of Lung Transplant Recipients at One Year Postoperatively

The correlation analysis showed that the Duke Activity Index score had a significant positive correlation with the preoperative BMI of lung transplant recipients (*r* = 0.489) and the duration of postoperative ICU stay (*r* = 0.340), whereas a significant negative correlation was found with the postoperative period until the Barthel score was > 60 (*r* = −0.335).

### 3.4. Multifactorial Analysis of Activity Status in Lung Transplant Recipients One Year After Surgery

In order to avoid the problem of multiple covariance among the factors, the factors that were correlated with the Duke index scale were diagnosed with covariance. The results showed that there was no covariance between the factors (Table 3).

**TABLE 3:** Covariance diagnosis of model parameters in multiple linear regression analysis.

Parameter	Covariance diagnostics		*F*	Adjust *R* ^2^	DW test^c^
	Tolerances	VIF^b^			
Age	0.742	1.347	6.670^d^	0.439	1.923
Preoperative BMI	0.738	1.356			
Length of postoperative ICU stay	0.925	1.081			
BI score > 60^a^	0.872	1.147			

^a^Length of time between the end of the operation and the time of the event.

^b^Variance inflation factor.

^c^Durbin–Watson.

^d^ *p* < 0.001.

Multiple linear regression analysis was performed with the DASI score of postlung transplantation patients as the dependent variable, and age, preoperative BMI, length of postoperative ICU stay, and length of the interval between postoperative and Barthel score > 60 min as the independent variables. As shown in Table 4, the factors identified through multivariate analysis as independently associated with the Duke activity status within 1 year after surgery include age (*β* = −0.382, *p* = 0.026), the preoperative BMI of the patients (*β* = 0.565, *p* = 0.002), and the postoperative length of stay in the ICU (*β* = 0.425, *p* = 0.007). Due to the limitations of the small sample size in this study, these should be interpreted as relevant factors rather than definitive determinants.

**TABLE 4:** Multiple linear regression analysis of DASI scores.

Independent variable	Regression coefficient	Standard error	*β*	*t*	*p*
Constant	3.435	12.172		0.282	0.78
Age	−0.418	0.177	−0.382	−2.368	0.026
Preoperative BMI	2.034	0.583	0.565	3.489	0.002
Length of ICU stay^a^	0.059	0.020	0.425	2.940	0.007
BI score > 60^b^	−0.005	0.003	−0.212	−1.426	0.166

^a^Postoperative.

^b^Length of time between the end of the operation and the time of the event.

## 4. Discussion

This study is based on an exploratory survey of the activity status of lung transplant recipients within 1 year after surgery at a lung transplant center in western China. It is a small‐sample, single‐center study. We have attempted to comprehensively present the activity status of lung transplant patients within 1 year after surgery, filling the gap in current research on the long‐term activity status of lung transplant recipients in this region, and providing a certain reference basis for the postoperative activity management of this population.

Lung transplant recipients initiate early physical activity at a late stage in the postoperative period, demonstrate slow progress in returning to self‐care, and exhibit low activity levels at 1 year postoperatively. Zheng et al. documented limited early postoperative mobilization in their study of lung transplant recipients, with merely 16.5% achieving independent ambulation within 72 h after surgery [15]. It is noteworthy that the events encompassed in this study were not merely the act of leaving the bed; rather, they were indispensable in terms of both distance and time. This distinction may have contributed to the substantial discrepancy in findings compared to those reported in other studies. The present study revealed that lung transplant recipients had a protracted recovery period for their self‐care abilities following surgery. The mean duration for resumption of independent eating was 29.73 days, and independent washing and independent bathing required 32 and 58 days, respectively. Additionally, the BI exhibited a mean recovery period of 39 days to return to mild dependence after surgery. The DASI score at 1 year postoperatively was 23.84 ± 13.66, which was only half of the total score. Ulvestad et al. scored 22.1 ± 6.9 mL/ (kg‐min) in the assessment of VO_2_peak in 54 double lung transplant recipients after surgery [16], which was higher than the value of 19.37 ± 6.20 mL/(kg‐min) in the present study. This difference may be attributable to the method of assessment employed in the two studies. Specifically, the former study utilized the cardiopulmonary exercise test (CPET) to determine VO_2_peak, whereas the latter study extrapolated results from Duke scale scores. However, a consensus has emerged that early mobility in postlung transplant patients is suboptimal. Wickerson et al. found that the majority of recipients were sedentary for 3–6 months after lung transplantation [17], and Langer et al. compared the mobility of lung transplant recipients with that of healthy individuals at 1 year postoperatively and found that the number of steps, the amount of time spent standing, and the amount of time spent in moderate‐intensity activity decreased by 42%, 29%, and 66%, respectively, in the lung transplant recipients [18], and the amount of time spent in sedentary activities increased by 30% compared with that of the healthy population. Postoperative mobility in lung transplant recipients is suboptimal.

In addition to the patient’s own factors such as weakness and fatigue, poor coping skills of patients and their families in postoperative rehabilitation also contribute to poor postoperative mobility, including patients’ role transition barriers [19], caregivers’ challenges in terms of psychological, economic, and knowledge acquisition [20]. During the follow‐up of this study, 80% of the family members refused to allow the patients to do housework, believing that they should rest instead of doing activities after illness. This also reflects the urgent need to improve the effectiveness of health education and exercise instruction for patients and their families after lung transplantation. PANG et al. surveyed 34 physicians, 97% of whom had provided physical activity‐related instruction to transplant patients, but only 18% were very confident in the effectiveness of the instruction, and the lack of guidelines for home exercise after lung transplantation was identified as a major barrier to physical activity instruction for healthcare professionals [21].

The long duration of mechanical ventilation was also one of the reasons for the low postoperative DASI score (23.84). In this study, 8 lung transplant recipients underwent mechanical ventilation for more than 25 days after the operation. Although deep sedation was avoided as much as possible and mechanical ventilation did not delay postoperative rehabilitation exercise, mechanical ventilation still caused ventilator‐induced diaphragmatic dysfunction, and most lung transplant recipients were in end‐stage lung disease before surgery. There is chronic low‐grade inflammation, which can induce a systemic inflammatory response syndrome‐like state and impair muscle function. Early postoperative gastrointestinal motility disorder leads to insufficient nutrient intake, and negative nitrogen balance can aggravate muscle loss. These factors contributed to the poor mobility of the patients in this study at 1 year after surgery.

This study found that the older the age, the worse the mobility of lung transplant recipients at 1 year after the operation. With the development of population aging and the advancement of lung transplantation technology, more and more elderly patients have received lung transplantation. In China, the proportion of lung transplant recipients over 65 years old was 21.3%, 20.7%, and 27.5% in 2019, 2020, and 2021, respectively, showing a significant upward trend [22]. In 2022, 81.1% of patients on the U.S. lung transplant waiting list were over 50 years old [23]. Aging causes a gradual decline in body functions, including respiratory muscle strength, lung ventilation, and muscle strength. By analyzing 24, 820 lung transplant patients, Iguidbashian et al. found that younger patients had a higher survival rate within 5 years after surgery than older patients [24]. The median survival of lung transplant recipients aged ≥ 65 years was only 4.41 years and worsened significantly with age [25]. When the basic physical conditions of lung transplant recipients are similar, the older the patient, the slower the recovery process. Therefore, there is a need to emphasize the improvement of postoperative mobility in elderly patients.

In this study, preoperative BMI was found to be positively correlated with patients’ postoperative mobility. The mean preoperative BMI of the patients in this study was 19.72 kg/m^2^, which is much lower than the 23.8 kg/m^2^ of international adult lung transplant recipients [26]. Joseph et al. [27] found that patients with lower preoperative BMI were less likely to achieve BMI recovery within 1 year, and BMI recovery within 1 year after transplantation was associated with longer survival. Therefore, management of preoperative BMI is particularly important. In 2023, Michael et al. [28] found that low BMI increased the risk of postoperative mortality in lung transplant recipients. This showed that preoperative BMI management was very important for improving patient outcomes, and avoiding a low preoperative BMI is beneficial for the recovery of postoperative mobility in lung transplant recipients. Although our study found that preoperative low BMI was a risk factor for poor 1‐year activity after transplantation, we were unable to fully investigate the impact of high BMI or the potential nonlinear (U‐shaped) association because the number of overweight/obese patients in our single‐center cohort was limited. Future studies with larger sample sizes and multiple centers are needed to explore the complete relationship between preoperative BMI and posttransplant activity, including possible U‐shaped or J‐shaped patterns.

There was a positive correlation between the length of stay in the postoperative ICU and the Duke index score of the recipients 1 year after surgery in this study, which is inconsistent with other relevant studies, which have found that the longer the length of stay in the postoperative ICU, the worse the postoperative muscle strength and recovery of patients [29, 30].

This study conducted an in‐depth analysis of the data and found that the reasons for this result might be as follows: Firstly, the recipients in this study began their rehabilitation on the second day after the surgery. Even in the ICU, their rehabilitation process was not affected. After discharge, patients who are still unable to walk to the bedside will be transferred to rehabilitation institutions to continue their rehabilitation process. In contrast, patients with short ICU stays were often discharged directly home without a formal rehabilitation plan. The proportion of patients who spent a long time in the ICU and continued to receive professional rehabilitation training after discharge was as high as 78% (a total of 9 patients had an ICU stay of more than 5 days after surgery, and 7 of them were transferred to rehabilitation institutions to continue their rehabilitation). Blanjean et al. found that patients with active physical activities in the ICU had significantly better muscle strength recovery ability 1 month after discharge than those who were less active [31]. Early and uninterrupted rehabilitation in the ICU might have improved the postoperative activity abilities of these patients. Secondly, this study only included patients with a survival time of at least 1 year. Patients who have been long‐term residents in the ICU might represent a more resilient subgroup. Patients with complex disease courses, death, or residual disabilities were excluded from the 1‐year assessment. Therefore, this association should not be interpreted as indicating that a longer ICU stay has a protective effect.

Postoperative activity data in this study were derived from nursing records and caregiver activity logs, although they were verified by nurses through weekly telephone calls. However, this approach still carries a high risk of measurement bias and inconsistency. These omissions limit the ability to draw reliable conclusions from the data.

The most significant statistical limitations of this study are the small sample size, as well as the gender imbalance, thus increasing the risk of model overfitting, unstable estimates, and type II errors. The significant imbalance in the gender ratio (90% male) in our study population reflects two factors: (1) the potential etiological situation among end‐stage lung disease patients receiving transplants in our region, where COPD and interstitial pulmonary fibrosis (both of which are more common in men, accounting for over 80% of all indications) are the main causes; and (2) the predominance of male patients during our study period. However, we acknowledge that this imbalance is extremely serious and limits the applicability of our research results to female recipients. In the future, we urgently need to conduct multicenter and large sample studies with an equal gender ratio to determine whether similar predictors also apply to other patients.

Furthermore, due to the concern of lung transplant patients about the risk of lung infection, many patients refused to undergo activity status assessment at the hospital. Therefore, in this study, only the DASI scale was used for activity status assessment. The patient research process included two stages: hospitalization and home care. Thus, the data collection involved different time points, and sometimes, if the data were not recorded in a timely manner, the recorder needed to relay on recall. These were important factors that may lead to data bias. However, this study improved the reliability of the data as much as possible by arranging dedicated personnel to collect and organize the data, confirming the data during home care with both the caregiver and the patient, and conducting frequent and scheduled follow‐up opportunities. Future research should conduct a comprehensive and objective assessment of the movement activity status of lung transplant patients from more dimensions such as nutritional status, muscle strength level, and the 6‐min walk test (6MWT), in order to present the postoperative activity status of lung transplant patients more comprehensively.

## 5. Conclusion

Our exploratory data suggest that the measures to enhance patients’ optimal activity status 1 year postoperatively should involve prioritizing the management of recipients older than 60 years of age, emphasizing preoperative BMI management among the diverse management components, and expediting the promotion of patients to regain their self‐care as soon as possible postoperatively to improve their long‐term postoperative mobility. This study is a small‐scale, single‐center research cohort with a significant gender imbalance. Moreover, physical activity was assessed through a self‐reported DASI scale rather than objective indicators such as the 6MWT or CPET. Therefore, the generalizability of the results needs to be verified in a larger scale cohort with more objective diagnostic indicators.

## Author Contributions

Xin Chen and DingDing Zhang designed and conducted the study. Xin Chen, JianQiong Gao, Tian Xu, XiangLing Yue, and ChunHan Cui were involved in sample processing and analysis. Xing He performed the statistical analyses and drafted the manuscript. DingDing Zhang critically reviewed the manuscript for important intellectual content and approved the final version to be published.

## Funding

This work was supported by Science and Technology Bureau of Chengdu (2026YF05‐00958‐SN), Chengdu Municipal Health Commission (2024025) and the Key Research and Development Program of Sichuan Provincial Science and Technology Department (2023YFS0311).

## Disclosure

All authors have read and approved the final version of the manuscript.

## Ethics Statement

The study was approved by the Ethics Committee of Sichuan Provincial People’s Hospital (2025188). Patients provided written informed consent.

## Conflicts of Interest

The authors declare no conflicts of interest.

## Supporting Information

Additional supporting information can be found online in the Supporting Information section.

## Supporting information


**Supporting Information** Supporting Table 1: Analysis of the differences in characteristics among recipients of different age groups.

## Data Availability

All data generated or analyzed during this study are included in this article. Further enquiries can be directed to the corresponding author.
