# Supplementary material for: Physical Activity Status and Related Factors of Lung Transplant Patients Within One Year After Surgery: A Single‐Center Observational Study
Source: Can Respir J. 2026 Aug 24;2026:5036015. doi: 10.1155/carj/5036015 (PMC13501507; doi:10.1155/carj/5036015)
Supplement: Supplementary file 1 [file CARJ-2026-5036015-s001.docx]

| Supplementary Table 1 Analysis of the differences in characteristics among recipients of different age groups | | | | |
| --- | --- | --- | --- | --- |
| Variables | ＜60 years  (n = 15) | **≥** 60 years  (n = 15) | t/Z | P |
| BMI(kg/m^2^) | 19.73±4.76 | 19.71±2.68 | 0.014 | 0.989 |
| Length of stay in hospital (h)^a^ | 1152.0[672.0-1512.0] | 1440.0[840.0-1848.0] | -0.996 | 0.319 |
| Barthel Index Score^b^ | 65 [45, 90] | 65[45, 75] | -0.333 | 0.739 |

Supplementary Table 1 Analysis of the differences in characteristics among recipients of different age groups
